# Uncertainties in Visual Observations of Floating Riverine Plastic

**DOI:** 10.1021/acsestwater.5c00223

**Published:** 2025-06-06

**Authors:** Paul Vriend, Thijs Bosker, Yvette Mellink, Frank Collas, Felipe Moscoso Cruz, Nadieh Kamp, Sylvia Drok, Martina G. Vijver, Tim H. M. van Emmerik

**Affiliations:** † Institute of Environmental Sciences, 84813 Leiden University, 2300 RA Leiden, The Netherlands; ‡ 69643 Rijkswaterstaat, Ministry of Infrastructure and Water Management, 2515 XP The Hague, The Netherlands; § Aquatic Ecology and Water Quality Group, 4508 Wageningen University and Research, 6708 PB Wageningen, The Netherlands; ∥ Department of Environmental Science, Radboud Institute for Biological and Environmental Science, Radboud University, 6525 AJ Nijmegen, The Netherlands; ⊥ Cubecumber, 4811 AB Breda, The Netherlands; # Hydrology and Environmental Hydraulics Group, Wageningen University and Research, 6708 PB Wageningen, The Netherlands

**Keywords:** litter, water surface, water quality, marine debris, hydrology, Rhine, Meuse, optimization

## Abstract

Accurate and reliable monitoring data are crucial for the design of effective plastic pollution reduction and mitigation strategies. One common approach to monitor macroplastic (>0.5 cm) in rivers is the visual observation method, where floating plastics are counted from bridges to estimate plastic flux. However, this method lacks robust uncertainty analyses, resulting in unknown error margins and potentially suboptimal monitoring strategies. The goal of this study was to quantify these uncertainties. Three key design elements that contribute to uncertainty include (1) cross-sectional coverage, (2) observation time, and (3) observation frequency. Through a case study on the Dutch Rhine-Meuse delta, we show how these uncertainties can be quantified and that they can be used to make informed monitoring design decisions. We further demonstrate that the detection rate of true flux (recovery rate) is a key parameter to consider during uncertainty analyses. By integrating an uncertainty optimization step into the design process, the efficiency and effectiveness of monitoring protocols can be improved. These insights enhance data quality and reliability, ultimately supporting efforts to mitigate the environmental impacts of macroplastic pollution.

## Graphical abstract

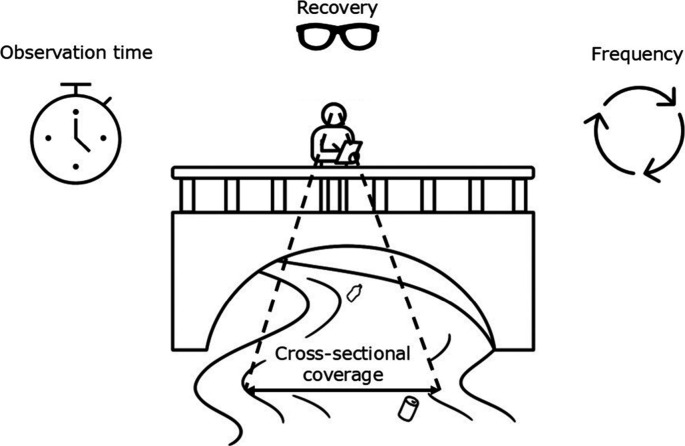

## 1 Introduction

Rivers play a crucial role in the global distribution of plastic pollution. An estimated 0.8–2.7 million metric tons of macroplastic pollution is discharged from rivers into oceans annually.^1^ This figure is expected to increase as plastic waste growth outpaces mitigation efforts.^2^ Negative effects of plastic pollution include reduced ecosystem functioning, damage to biota, adverse economic effects, increased flood risk, and potential implications for human health.^3,4^ High-quality observational data on plastic pollution, obtained through long-term monitoring, are essential for developing effective reduction strategies.^37^ These data underpin the design and evaluation of policy measures and the validation of models that map pollution pathways and material flows through the environment.^5–7^

The visual observation method, where floating macroplastics (>0.5 cm) are observed from bridges, has been widely used to monitor macroplastics floating on the water surface.^8,9^ Initially developed during the RIMMEL project,^10^ the method has undergone several adaptations, for example, through the inclusion of net sampling to allow for more detailed characterization of litter,^11^ making alterations to allow for more rapid application of the method,^12^ and by optimizing how observations are executed.^13^ Visual observations have been used to quantify macroplastic pollution at different spatial scales such as single locations,^14^ river basin scale,^15,38^ country scale,^16^ and continental scale.^9^ The visual observation method is often preferred over other methods (e.g., net sampling or the use of cameras) because it requires minimal technical skills, is easy to scale up, involves little training, and is highly flexible in its use.^8,17,18^ However, research on improving the quality of monitoring data collected with this method is lacking.^19^

Design choices for the implementation of the visual observation method are central to monitoring strategies, yet their effects on data uncertainties have yet to be analyzed fully. One such design choice is cross-sectional coverage or the section of the river width observed from the bridge. The original RIMMEL protocol recommends a single observer positioned centrally on the bridge to monitor floating macroplastics.^10^ For wider rivers, the bridge is often divided into segments, each observed separately, and then extrapolated to represent the full cross section.^11^ The number and width of these segments determine the cross-sectional coverage.^39^ It is essential to optimize the cross-sectional coverage as the horizontal distribution of floating is not uniform or constant over time.^16^ Another key element is observation time, the duration over which macroplastic items are counted, which vary widely among studies (typically ranging from 1 to 60 min; e.g., refs 20 and 21). Plastic flux is often reported in items per hour. Observed counts within a shorter observation period are therefore extrapolated linearly, introducing uncertainty.^40^ The observation frequency depends on the specific research objective. For instance, to quantify annual transport in a river system, frequent observations are necessary to cover annual variations that occur (e.g., ref 16), whereas measuring macroplastic flux at a single point in time may require only one round of observations (e.g., ref 13). Next to study design, the recovery rate, the proportion of the true plastic flux that is detected, needs to be accounted for.^19^ Smaller macroplastic items are more challenging to observe, and without accounting for recovery rates in data analysis, estimates of macroplastic flux may be biased.^19^

Monitoring campaigns are often a compromise between the need for optimal coverage and limited resources.^36^ Quantifying the uncertainties associated with design choices can help guide more effective monitoring campaigns by linking monitoring effort to specific research goals.^22^ Understanding the uncertainties helps prioritize improvements and identify when further investment in this element offers diminishing returns on data quality. This systematic approach we present here will help to develop tailored monitoring strategies, enhancing data quality and reducing overall costs.

Here, we quantify uncertainties arising from various design choices within visual observation monitoring protocols. Our paper will support practitioners in optimizing monitoring approaches by balancing data uncertainty, research objectives, and resource constraints. We use the example of the Dutch Rhine-Meuse delta to demonstrate how it can be integrated to enhance data quality, thereby supporting improved modeling efforts and more informed policy design.

## 2 Methods

### 2.1 Site Characterization and Monitoring Data

The study area includes the Dutch sections of the Rhine and Meuse rivers (Figure 1). The catchments of these rivers are densely populated and industrialized.^23^ The River Rhine enters The Netherlands 161 km upstream from the river mouth. At 147 km, the Rhine bifurcates into the Waal (67% of the discharge), Nederrijn (22%), and IJssel (11%).^24^ The Waal and Nederrijn then converge 42 km upstream from the river mouth. The Meuse enters The Netherlands 250 km upstream from its river mouth. Its discharge is highly regulated using weirs and is about 10% of the mean discharge of the Rhine system (230 and 2200 m^3^/s, respectively^25^). Near the river mouth, the branches of the Rhine and Meuse systems merge and intermingle. The IJssel river drains into Lake IJssel after 125 km.

**Figure 1:**
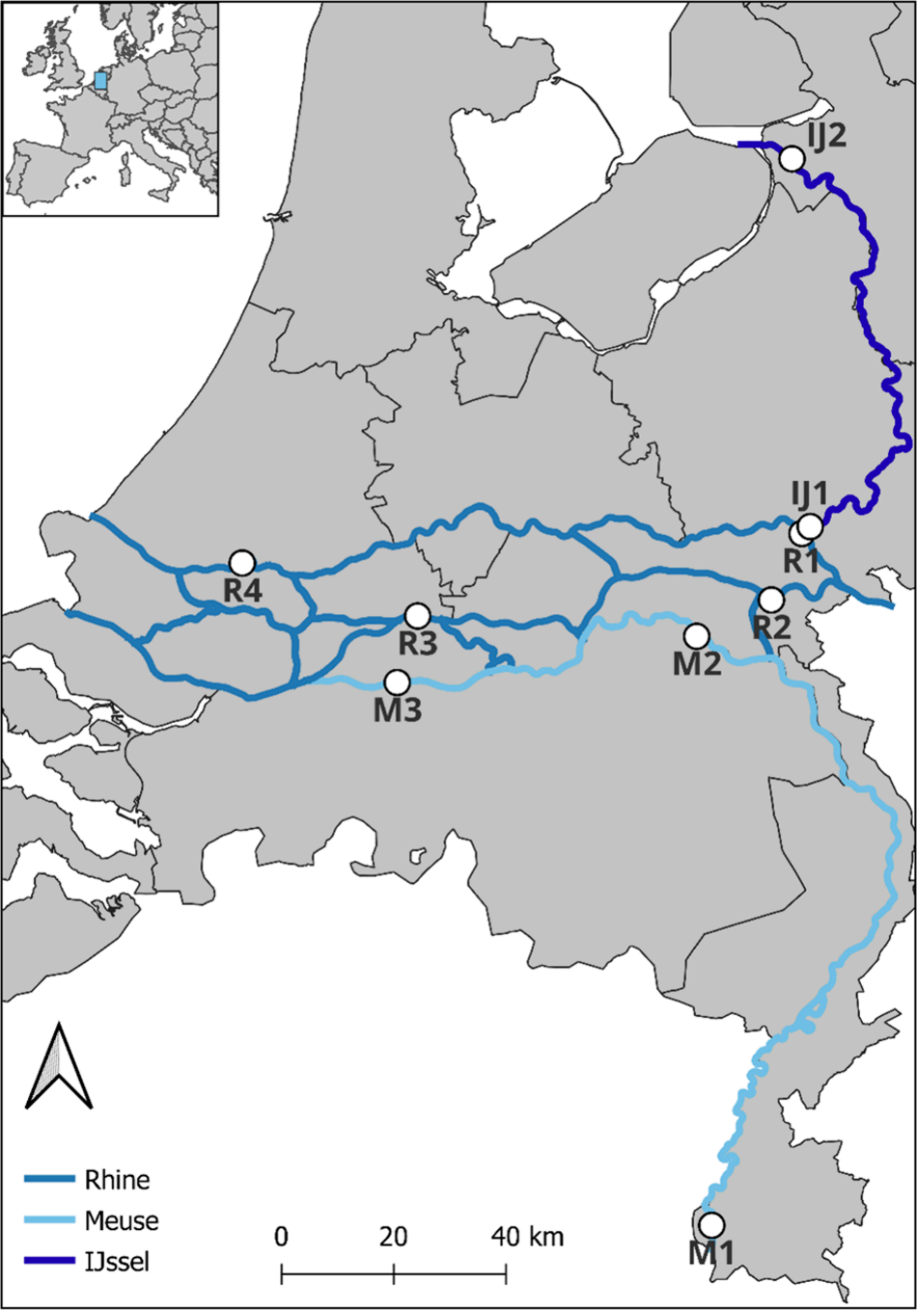
Monitoring locations used to collect data on floating macroplastic pollution in the Rhine (R1–4), Meuse (M1–3), and IJssel (IJ1–2) rivers. Location R4 is the tidal zone of the Rhine delta.

Visual observations were conducted at a monthly frequency from January to December 2023, following the protocol by Mellink et al.,^26^ which is an adaptation of the RIMMEL protocol.^9^ Observations were performed at nine bridges (one tidal (R4) and eight nontidal) crossing the Rhine, Meuse, and IJssel rivers (Figure 1). Observation sites were selected to include entry points of each river into The Netherlands and points near the estuary. Each bridge was divided into three to six segments depending on the river width, with segment width determined based on bridge height and the observer’s field of view, as recommended by Kuizenga et al.^13^ This approach resulted in cross-sectional coverage ranging from 22% to 97% (Supporting Information A). The observation time for each segment was 20 min.

Plastic flux *F* (number of items per hour) at each location was calculated using eq 1, which uses a combination of flux observed at each segment in items *f*_*i*_ (number of items per hour), the width of that segment *w*_*i*_ (meters), the river width at the specific location *W* (meters), and the total number of segments on the bridge *S*.

1 $$F=W\overset{s}{\underset{i=1}{\sum }}\left(\frac{f_{i}}{w_{i}}\right)/S$$

### 2.2 Uncertainty Analyses

#### 2.2.1 Cross-Sectional Coverage

Achieving full cross-sectional coverage is challenging due to resource constraints, particularly in wider rivers.^8^ As a result, monitoring often involves observing part of the river and extrapolating these observations to the full width.^11^ We quantified the uncertainty introduced when cross-sectional coverage is reduced, increasing the extrapolation factor for calculating the total plastic flux.

We first calculated the total plastic flux for each month per location, using eq 1. We assumed that these values are the true values of the plastic flux. We then calculated the plastic flux based on a reduced number of segments, ranging from one to the total number of segments at that location. For each number of segments, we randomly drew a subset from the total population for that location and month and extrapolated these values linearly to the full river width to determine the extrapolated flux. This process was repeated 1000 times following a Monte Carlo simulation. For each subset, we compared the extrapolated total plastic flux with the true plastic flux. The difference between these was used to quantify the uncertainty associated with partial cross-sectional coverage. To allow for comparison between bridges with varying amounts of segments, the number of segments was converted to a percentage of the river width they represent (e.g., one segment is 20% of the river width when there are a total of five segments on the bridge). Results were combined and grouped into bins representing 20% intervals of coverage. Differences between the groups were statistically tested using a one-way Kruskal–Wallis test (with α set at 0.05). All statistical analyses were done in R version 4.3.3, using the ggplot2 library for the figures.

#### 2.2.2 Observation Time

The standard unit to report plastic flux is items per hour. While some studies have adopted a 60 min observation time (e.g., ref 9), most use shorter observation times (e.g., 1–20 min; ref 13). This analysis assessed the effect of reducing the observation time on the uncertainty levels in the collected data. We compared extrapolated plastic flux values based on observation times ranging from 1 to 59 min to the true plastic flux obtained with a 60 min observation time, where no uncertainty is introduced through extrapolation.

We collected additional data at locations R4 and IJ2 for 60 min to determine a true hourly plastic flux per segment. The exact time (HH:MM) of each observed item was noted, allowing for minute-by-minute item counts. Observations were repeated twice per segment, resulting in a total of 18 one-hour observation sessions across six segments at R4 and three segments at IJ2. Two replicates with zero items observed were excluded from further analysis, as the deviation between extrapolated and observed fluxes does not change with a change in the observation time. Next, for each of the 16 replicates, all unique combinations of consecutive time blocks ranging from 1 to 59 min were selected. Each of these blocks was then linearly extrapolated to estimate an hourly plastic flux. The relative difference of each extrapolated value from the true 60 min flux value was calculated for each replicate to determine the uncertainty introduced by using shorter observation times.

#### 2.2.3 Observation Frequency

The flux of floating riverine plastic varies significantly over time.^16,28^ The smallest uncertainty occurs when monitoring continuously, as this would fully capture this variability. However, resource limitations necessitate a limit on the frequency, increasing uncertainty. Monitoring results are often extrapolated to flux per year for subsequent use in scientific research and policy. For example, modeling studies to quantify the export of plastic to the marine environment use data on an annual time scale for validation purposes (e.g., refs 29 and 30), as do material flow analysis studies.^6^

We determined the effect of reducing the number of observations per year on the estimated floating plastic flux. The full data set includes 12 observations taken at monthly intervals. We calculated the annual mean transport based on these data and assumed this to be the true annual transport. For this, we used linear extrapolation as the relationship between flux and discharge was found to be not significant (Supporting Information C). Next, we randomly drew subsets of observations using fewer data (1–11 months) and calculated the yearly flux based on these subsets. This was repeated 1000 times per subset size. We then calculated the difference between the true flux and the extrapolated flux.

#### 2.2.4 Recovery Rate

As smaller plastic items are harder to detect, observers may not detect all floating macroplastic items of 0.5 cm and larger.^31^ The ratio of detected to true flux is the recovery rate.^19^ Monitoring protocols often set limits to the sizes of items included to reduce uncertainties. For example, the OSPAR beach litter monitoring protocol omits items smaller than 2.5 cm.^32,33^ However, since item sizes are often estimated and not measured during visual observation,^41–43^ items of smaller, uncertain size cannot be excluded, resulting in residual uncertainty. We estimated how much of the true macroplastic flux may be missed during visual observations by (1) estimating recovery rates under controlled conditions for different items sizes, item colors, and bridge heights, (2) constructing a particle size distribution of macroplastics in the Rhine-Meuse delta, and (3) calculating a correction factor for our case study by combining the found recovery rates and the relative share of each size in the item size distribution.

We generated a data set to assess recovery rates across different item sizes. Items of known size and color were released from a vessel at distances of 1–5 m upstream from four bridges (IJ1, height of 14 m; IJ2, height of 5 m; R1, height of 21 m; R2, height of 19 m). Observers located on the downstream side of the bridge counted the items as they floated by. Three observers were stationed at the same location on the bridge, separated by barriers so they could not see each other. This was done to prevent them from visually influencing each other’s observations. The observers were instructed by an experienced observer to ensure the uniform execution of the monitoring protocol. The items used in the experiment were made from a 9 mm thick cork sheet, sized at 3 cm × 3 cm, 2 cm × 2 cm, and 1 cm × 1 cm, and colored brown, blue, black, red, and white. Cork was chosen to ensure that the items were floating on the surface when passing the observers. Colors and sizes were selected based on expert assessment to best represent items typically observed in floating debris monitoring. Transparent items were excluded from release following permit regulations. Items were released in batches of a known amount ranging from 22 to 45 particles of the same size over a 1 min interval, ensuring that particles did not stick together. For the sake of efficiency, two batches of different colors but identical sizes were released together. Across all sites, 7677 particles were released in 164 batches, including 2782 particles of 9 cm^2^ (3 cm × 3 cm, 92 batches), 2774 particles of 4 cm^2^ (2 cm × 2 cm, 92 batches), and 2121 particles of 1 cm^2^ (1 cm × 1 cm, 75 batches). The recovery rate was determined by calculating the ratio of counted items to released items.

To examine the combined effect of size, color, and height on recovery rates, statistically significant differences between groups were assessed using Schreier–Ray–Hare tests (α = 0.05). No significant interactions were found for color and size (*P* = 0.258) or height and size (*P* = 0.966 (Supporting Information D)), so Kruskal–Wallis tests (α = 0.05) were performed on size, color, and height individually, followed by Dunn’s post hoc tests to identify specific group differences.

A second data set was collected providing a particle size distribution for floating macroplastic in the Rhine-Meuse delta. For this data set, samples collected by Vriend et al.^34^ and Oswald et al.^35^ were reanalyzed using camera imagery and a scale in order to determine the area and mass of each particle. Both studies used trawl nets with a 4 m wide, 1 m tall opening and a mesh size of 0.5 cm. Only the surface net samples from these studies were used, as these are most representative for floating plastics as these sampled floating particles and particles up to 1 m depth. Particles were individually photographed using a IRIScan Pro 6 scanner, and the mass was taken using a scale with a 0.01 mg accuracy. The surface area of each particle was determined by drawing polygons around the circumference of the picture (ImageJ version 1.54i). A total of 1275 particles were analyzed with sizes ranging from 0.06 to 99.1 cm^2^ and masses ranging between 0.1 and 1208.1 mg.

The recovery rates per size class and their relative share in the particle size distribution were used to estimate the share of the true flux potentially missed during the visual observation. A correction factor (*C*) was calculated using eq 2, based on the found recovery rate of each item size (*R*_*i*_ in percent) and the relative share (*p*_*i*_ in percent) this size has in the particle size distribution. This correction factor was then used to compare a hypothetical observed flux to the true flux. To determine the recovery rates per size group, <1 cm^2^ items were grouped with the 1–4 cm^2^ group. We note that differences in recovery rates per color were not included in this correction factor as a data set on the color per size class was lacking, and transparent items had to be excluded from the recovery data set due to regulatory restrictions.

2 $$C=\sum \frac{p_{i}}{R_{i}}$$

## 3 Results

### 3.1 Cross-Sectional Coverage

Lower cross-sectional coverage significantly increases the uncertainty of estimating the true plastic flux (*P* < 0.001 (Figure 2)). For a cross-sectional coverage >61%, the median deviation is 0. Between 41% and 60%, the median deviation drops to −4.8%. For cross-sectional coverages ranging between 21–40% and <20%, the median values decrease further to −16.7% and −50.0%, respectively. These results indicate that in our case study, using a cross-sectional coverage of less than 60% to estimate plastic flux will likely result in an underestimation. Moreover, the risk of deviation from the actual mean increases, as demonstrated by an increase in the interquartile range (IQR), which increases rapidly with a reduction in cross-sectional coverage. For coverage levels exceeding 80%, the IQR is 29% (Figure 2). In contrast, if <20% of the bridge is used, the IQR increases to 143%, meaning that there is a change of substantial under- or overestimation of the total flux.

**Figure 2:**
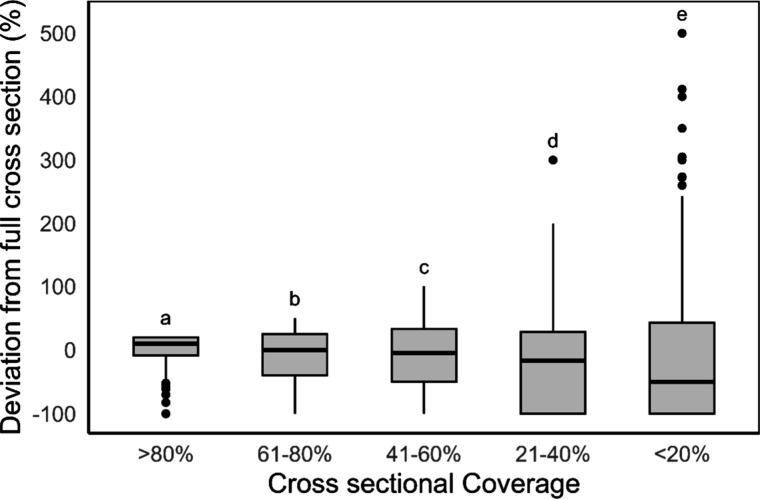
Deviation of extrapolated plastic flux in the Rhine-Meuse delta based on a subsection of the cross sections compared to plastic flux based on full cross-sectional coverage of the river system (based on a Monte Carlo simulation with 1000 repetitions, where observations for all locations are combined). Letters indicate the results of a one-way Kruskal–Wallis test.

The decrease in the median deviation in our case study and the increase in IQR are likely driven by instances where no litter is observed. As cross-sectional coverage decreases, the probability of extrapolating flux values based on a subset where all segments have zero observed flux increases. Similarly, extreme overestimates (>250%) also become more frequent with reduced cross-sectional coverage, driven by the overextrapolation of single high observations. Observations without any litter are more frequent than high observations in the observational data set.

Linear extrapolation to the full cross-sectional coverage introduces uncertainty because the horizontal distribution of floating macroplastics in the Rhine-Meuse delta is not uniform. These findings can inform decisions on the optimal cross-sectional coverage during monitoring. Two key changes in the IQR can guide the choice of coverage level. The first major shift in IQR occurs between the >80% and 61–80% groups. Beyond this, the IQR remains relatively stable until a substantial increase occurs for the 21–40% group. Therefore, a monitoring strategy in the Rhine-Meuse delta could aim for cross-sectional coverage of more than 80%, resulting in an uncertainty level in the IQR of 29%. If this level is unattainable, coverage could be reduced to 41–60%. Whether this uncertainty is acceptable depends on the goal of the monitoring strategy and on the uncertainties introduced in the other design choices.

### 3.2 Observation Time

During our observation period, the observed flux ranged from 0 to 23 items per segment per hour (Supporting Information B). Based on our simulations, we show that the mean 95% confidence interval (CI) of the deviation between extrapolated and observed flux across time follows a distinct three-phase pattern (Figure 3). Visual inspection shows that between 57 and 35 min, the deviation remains relatively constant and low, ranging from 5% to 10%. This is followed by a linear increase, reaching a mean of 57% at the 10 min mark. In the final phase, the deviation increases exponentially, with a deviation of 77% at the 5 min mark and peaking at 165% at the 1 min observation time. A similar three-phase trend is observed when focusing on the maximum deviation values. In our case, using a 35 min observation time may be optimal for minimizing uncertainty. This is different from the protocol used to collect the monitoring data used for our case study, where the observation time was set to 20 min.

**Figure 3:**
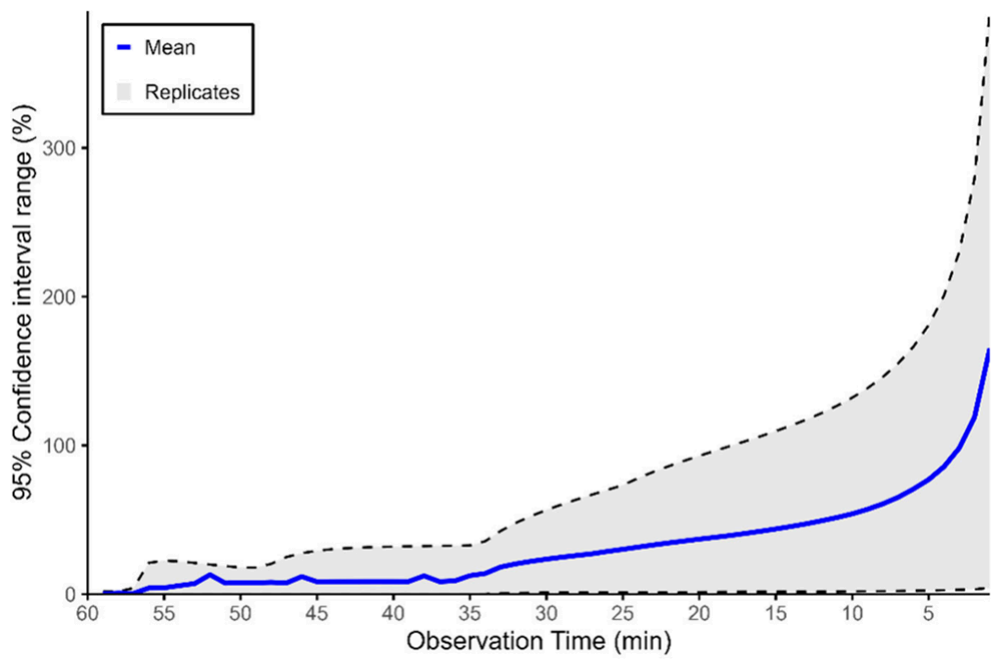
Range of the 95% confidence interval for the deviation of extrapolated hourly flux based on all unique combinations of 1–59 min, compared to the actual hourly transport over 60 min. The blue line represents the mean deviation across all replicates from both locations, providing an average trend in extrapolated flux accuracy. Gray dotted lines denote the minimum and maximum values of all individual replicates.

### 3.3 Observation Frequency

The mean 95% confidence interval (CI) of the deviation between extrapolated and observed flux across time reveals a two-phase pattern that can guide the determination of the optimal observation frequency. Between 11 and three observations, the deviation increases linearly, while in the range of three to one observation(s), the increase is exponential. The mean 95% CI range increases from 2% with 11 observations to 26% when extrapolated based on a single month. Similarly, the spread in the replicates increases from 4% with 11 observations to 21% with just one observation. This pattern suggests that the greatest reduction in uncertainty occurs when the frequency increases from one to three observations per year (Figure 4). Beyond this frequency, each additional observation contributes approximately a 3% reduction in uncertainty.

**Figure 4:**
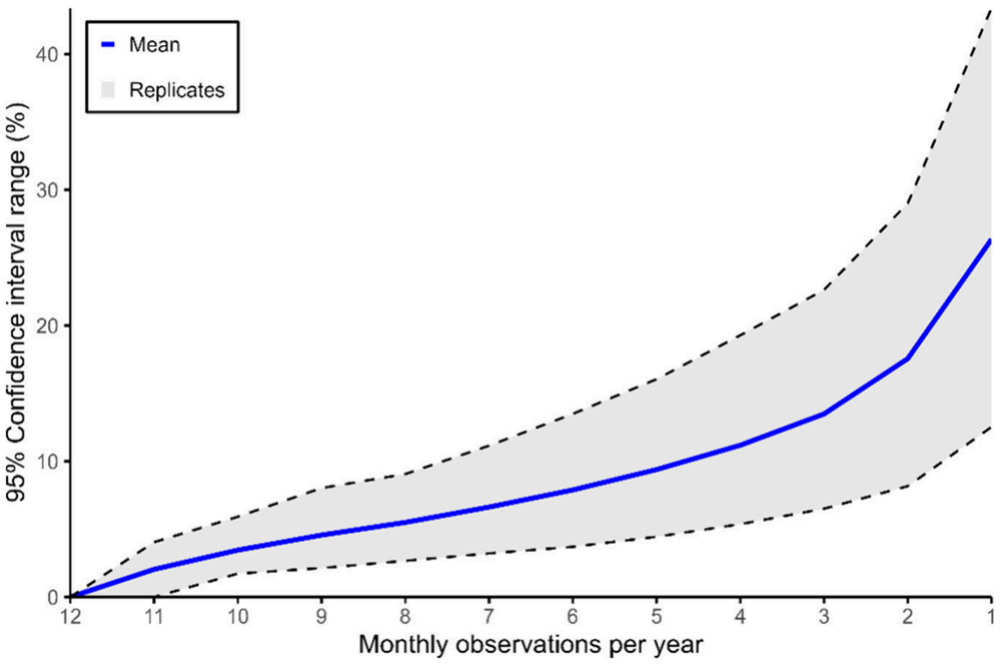
Range of the 95% confidence interval for the deviation in extrapolated annual transport (in percent) based on a Monte Carlo simulation of 1–11 observations compared to 12 observations. The blue line represents the average deviation across all locations, and dotted lines show the maximum and minimum values across all locations.

### 3.4 Recovery Rate

We find that the size and color of floating macroplastic significantly influence recovery rates (Figure 5). The differences in mean recovery rates across size classes were significant, with values of 83.2% (*P* < 0.001) for 9 cm^2^ items, 68.5% (*P* < 0.001) for 4 cm^2^ items, and only 22.9% (*P* < 0.001) for 1 cm^2^ items (Figure 5A). When considering the influence of color, recovery rates were higher for brown and white items (76.6% and 67.4%, respectively) compared with red (55.0%), black (48.6%), and blue (43.2%) items (Figure 5B).

**Figure 5:**
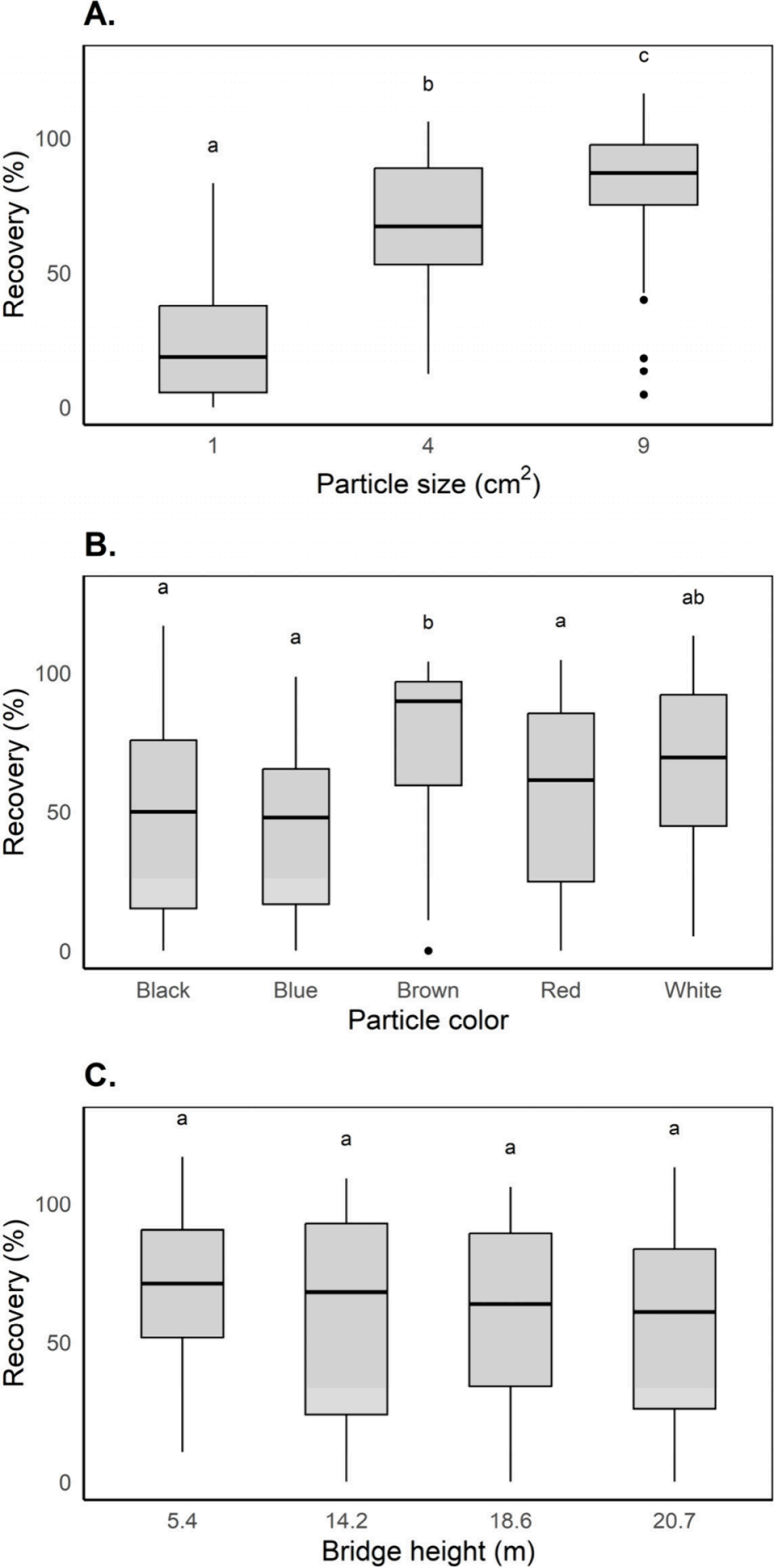
Found recovery rates in percent (A) per particle size class (square centimeters), (B) per particle color, and (C) per bridge height (meters), with lowercase letters indicating statistically significant differences.

The influence of the bridge height on the recovery is more nuanced. Considering just height and recovery (not including size) revealed no significant differences in recovery rates for the range of heights that were included in this study (Figure 5C). However, we do see that recovery rates are lower for smaller items and taller bridges (Supporting Information D) and that significant differences are present when examining the combined effects of size and height for extreme cases (low bridge height and large particles vs high bridge height and small particles (Table D6)). We therefore expect that height will significantly influence recovery rates with bridges taller than the range tested and with smaller item sizes. We suggest that these experiments be repeated with taller bridges, a larger variety of item sizes, and item shapes more representative of actual litter. With these results, it remains unclear how to effectively account for the decreasing recovery rates with increased heights and smaller items. Therefore, we do not account for this in the correction factor and emphasize that further research should explore the relationship among distance, item size, and recovery.

Our particle size distribution data indicate that while most of the items are small (<4 cm^2^ items represent 66.8% of the population), the majority of the total mass is concentrated in the larger particles (78.3% of the mass is concentrated in >4 cm^2^ items). The size distribution and cumulative mass percentage are shown in Figure 6, with the blue, red, and gray bars highlighting the 1, 4, and 9 cm^2^ size classes, respectively, matching the size of the particles that were released from the vessel to find the recovery rates. Items smaller than 4 cm^2^ comprise 66.8% of the item count (852 items) but only 21.7% of the total mass. Items in the 4–9 cm^2^ range make up 12.4% of the item count (184 items) and 11.1% of the mass. Items larger than 9 cm^2^, comprising 18.7% of the count (239 items), contributed the largest share of the total mass (67.1%).

**Figure 6:**
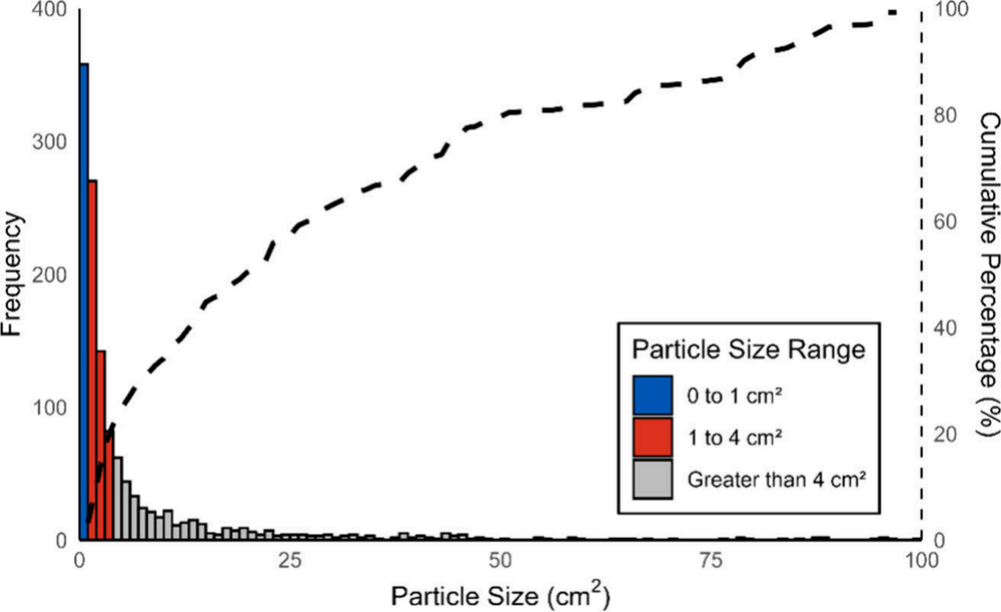
Particle size distribution plot of the macroplastic (>0.5 cm) items sampled in the Rhine river, with size frequency on the primary axis (histogram) and cumulative mass for these size classes on the secondary axis (dotted line). The colors correspond with the size of the items released during the recovery experiment: 1 cm^2^ (blue), 4 cm^2^ (red), and 9 cm^2^ (gray).

Combining the found recovery rates with the relative share of these items in the particle size distribution reveals a correction factor of 3.3 for our case study. This indicates that for an observed flux of 100 items per hour, the true flux of macroplastic items (>0.5 cm) is approximately 330 items per hour; the observed flux is therefore 30.3% of the true flux. In addition, this correction factor likely underestimates the true value, as transparent items were excluded from the recovery rate experiment. Based on observations during the net sampling campaign of Vriend et al., a majority of plastic particles in the Rhine-Meuse delta (>80%) are transparent, which likely complicates visual detection from bridges. Moreover, it is important to note that these findings primarily apply to item counts. If the objective is to estimate the mass flux of floating plastics, the focus shifts toward larger items, which account for the majority of the total mass. As shown in Figure 6, >4 cm^2^ items comprise 78.8% of the total mass with a minimum recovery rate of 68.5%. However, converting item counts to mass remains challenging due to the limited availability of consistent size-to-mass data for floating plastics.

## 4 Discussion

### 4.1 Implication for Monitoring Design and Optimization

Our results illustrate the importance of quantifying uncertainties in design choices before starting long-term monitoring projects. For our case study, uncertainties introduced through the different design elements can introduce up to 400% deviation each in extreme cases. However, the expected mean deviation varies substantially between the different elements (e.g., 29–143% for cross-section coverage, 0–165% for observation time, and 0–26% for observation frequency).

Our results also help optimize design choices for uncertainty. First, a high-resolution data set should be collected, which can be used to scale down to the optimal form. Using this data set, logical cutoff points can be identified for each design element, after which diminishing returns are reached. For example, in our case study, we would increase the observation time to 35 min, increase the cross-sectional coverage to 80%, and decrease the observation frequency to four to six observations. Ignoring these insights can lead to costly or suboptimal monitoring strategies. We note that the uncertainties found are context-specific and may not apply to other rivers; therefore, repeating these experiments in other rivers is important. Context-specific factors that may influence the results include changes in flow rates, seasonal variations, bridge height outside of those tested in this study, plastic characteristics, and segment location. For example, an observation frequency of four times per year may be enough to capture plastic flux under average discharge conditions but has a high chance of missing higher plastic transport rates during high-discharge events. The annual mean may therefore come with a larger uncertainty.

### 4.2 Importance of Recovery Rates in Flux Estimation

Our findings highlight the importance of accounting for recovery rates when estimating riverine plastic, a factor often overlooked in previous studies.^19^ The key issue is that no definitive lower size limit can be set for items that are included in the observations as the size of items it not measured when counting. We show that smaller items are counted with a significantly lower recovery rate. This has a substantial impact when quantifying plastic flux in terms of item count but is less influential when assessing total mass, as most of the mass is concentrated in larger particles, which have a higher recovery rate. We estimate a correction factor of 3.3 for converting observed flux to the true flux in the Rhine-Meuse delta. This figure is likely an underestimate, as transparent items were excluded from our experiments, which are likely harder to detect and represent a large portion of the total plastic flux (>80%, based on observations during sampling). Even though the correction factor can be refined further by including color and bridge height in the equation, these findings suggest that previous studies (e.g., refs 9 and 13) may have underestimated fluxes by not including recovery rates, underscoring the need for improved methodologies and greater standardization in future studies. Uncertainty analyses were integrated into monitoring workflows.

### 4.3 Integrating Uncertainty Analyses into Monitoring Workflows

Finally, we emphasize the importance of incorporating uncertainty optimization analyses, such as the one conducted in this study, into the workflow of monitoring design. In recent years, several efforts have been made to standardize the process of developing monitoring protocols (e.g., refs 8, 22, and 36). These studies generally propose a cyclical workflow that includes (1) identifying research goals, (2) considering river characteristics and available resources, (3) designing and implementing monitoring strategies, and (4) evaluating and reassessing monitoring goals.^36^ We propose adding an additional step to this cycle before implementing the monitoring strategy. In this step, the type of uncertainty analysis demonstrated in this study would be performed for the target location. The insights gained from these analyses then inform the final design of the monitoring strategy. By incorporation of this step, the resulting monitoring strategy can be better tailored to the specific context, making it more cost-effective and improving data quality.

## 5 Conclusions

Bridge-based visual observations are common for monitoring floating riverine macroplastics. Design choices influence the data uncertainty. We propose methods to quantify these uncertainties and apply them in the Rhine-Meuse delta case study. Our findings show that the key design elements cross-sectional coverage, observation time, and observation frequency can each introduce uncertainty, up to 500%, 400%, and 45%, respectively. We also identify thresholds for each element where the marginal cost of reducing uncertainty levels increases, which can inform decisions regarding monitoring strategy design.

We highlight the significance of accounting for recovery rates when estimating riverine plastic fluxes based on visual observations. The key concern is that no fixed boundary can be set for the lower size limit as the size of items it not considered when counting. We show that recovery rates can vary by 23–83% depending on size and color. This likely leads to an underestimate of the total floating macroplastic flux in rivers. We estimate the true macroplastic (>0.5 cm) flux could be 2-fold higher than initially counted in our case study. We emphasize the importance of repeating these experiments at more locations and to further improve this factor by including color and bridge height.

We emphasize the need for incorporating uncertainty analyses as proposed in this study into workflows for designing monitoring strategies. Without these analyses, there is risk that monitoring strategies are unoptimized. In these cases, resources are not spent efficiently to reduce uncertainties, negatively affecting data quality. We therefore suggest including uncertainty analyses into the workflow of monitoring design. As more countries are developing policies and strategies to minimize plastic pollution, this is urgently needed.

## Supplementary Material



## Notes

Segment width calculated for the nine monitoring locations included in this study, with the location, river width at that location, number of segments used, segment width, and total river width covered using the field of view + height method and the equidistance method, as well as the percentage difference in coverage between the two methods (Supporting Information A), number of observed items per minute per replicate during the observation time experiment (Supporting Information B), results of using different methods to extrapolate the observed plastic flux to the annual plastic flux (Supporting Information C), and found recovery rates per size, color, and height and the results of the Kurskal–Wallis test and subsequent Dunn’s post hoc tests (Supporting Information D) (PDF)
