# Supplementary material for: Uncertainties in Visual Observations of Floating Riverine Plastic
Source: ACS ES T Water. 2025 Jun 6;5(7):3920–8. doi: 10.1021/acsestwater.5c00223 (PMC12262360; doi:10.1021/acsestwater.5c00223)
Supplement: Supplementary file 1 [file ew5c00223_si_001.pdf]

# Uncertainties in visual observations of floating riverine plastic

*Paul Vriend[1,2*], Thijs Bosker[1], Yvette Mellink[3], Frank Collas[2,4], Felipe Moscoso Cruz[5], Nadieh Kamp[2], Sylvia Drok[2], Martina G. Vijver[1], Tim H. M. van Emmerik[6].*

[1]Institute of Environmental Sciences, Leiden University, Leiden, 2300 RA, the Netherlands
[2]Rijkswaterstaat, Ministry of Infrastructure and Water Management, 2515 XP, The Hague, the Netherlands
[3]Aquatic Ecology and Water Quality Group, Wageningen University and Research, 6708 PB Wageningen, the Netherlands
[4]Department of Environmental Science, Radboud Institute for Biological and Environmental Science, Radboud University, 6525 AJ, Nijmegen, The Netherlands
[5]Cubecumber, 4811 BR, Breda, the Netherlands
[6]Hydrology and Environmental Hydraulics Group, Wageningen University and Research, 6708 PB, Wageningen, the Netherlands.

**Corresponding Author**

*Paul Vriend, paul.vriend@rws.nl

**Supporting Information**.

**Supplementary Information A**: Segment width

**Supplementary Table A1.** Segment width calculated for the 9 monitoring locations included in this study. With the location, river width at that location, number of segments used, the segment width and total river width covered using the field of view + height method and the equidistance method, as well as the percentage difference in coverage between the two methods.

| | | | Segment width based on field of view | | Segment width based on equidistance | | |
|---|---|---|---|---|---|---|---|
| Location | River width (m) | Number of segments | Segment width (m) | Coverage (%) | Segment width (m) | Coverage (%) | Difference (%) |
| R1 | 120 | 4 | 29.0 | 24.2 | 30.0 | 25.0 | 3.4 |
| R2 | 330 | 5 | 26.0 | 7.9 | 66.0 | 20.0 | 60.5 |
| R3 | 500 | 6 | 21.3 | 4.3 | 83.3 | 16.7 | 74.5 |
| R4 | 480 | 6 | 17.9 | 3.7 | 80.0 | 16.7 | 77.6 |
| IJ1 | 75 | 3 | 19.9 | 26.5 | 25.0 | 33.3 | 20.5 |
| IJ2 | 205 | 6 | 7.6 | 3.7 | 34.2 | 16.7 | 77.9 |
| M1 | 165 | 6 | 10.7 | 6.5 | 27.5 | 16.7 | 61.0 |
| M2 | 135 | 5 | 19.3 | 14.3 | 27.0 | 20.0 | 28.4 |
| M3 | 265 | 5 | 16.0 | 6.1 | 53.0 | 20.0 | 69.7 |

**Supplementary Information B**: Observation time – observed items per minute per replicate

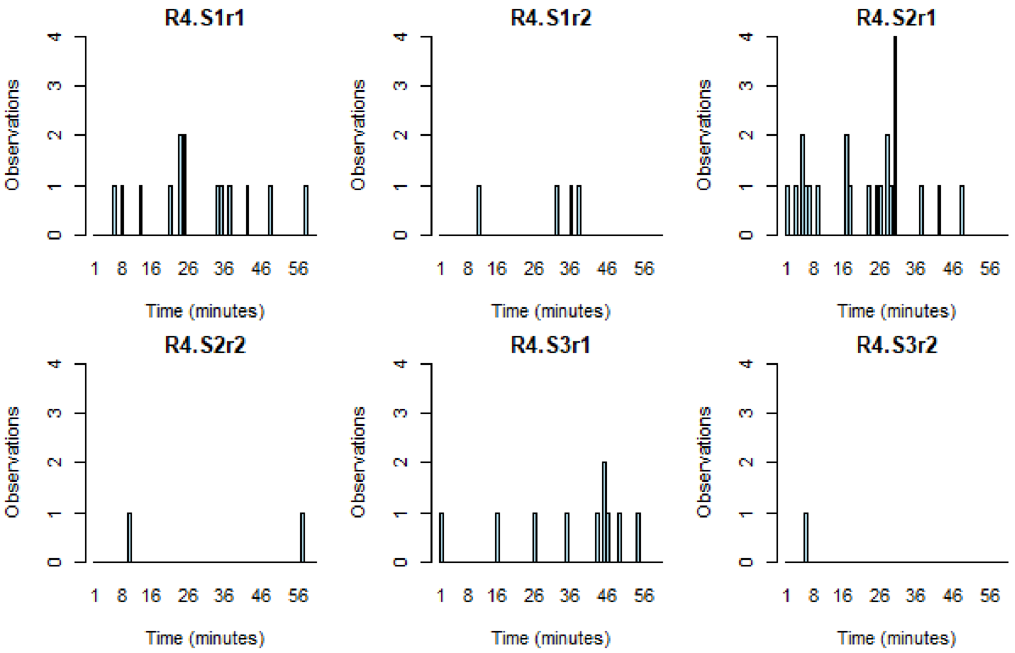


**Supplementary figure B1**. Observed items per minute for location R4, segments 1-3 (S) and replicates for that section (r).

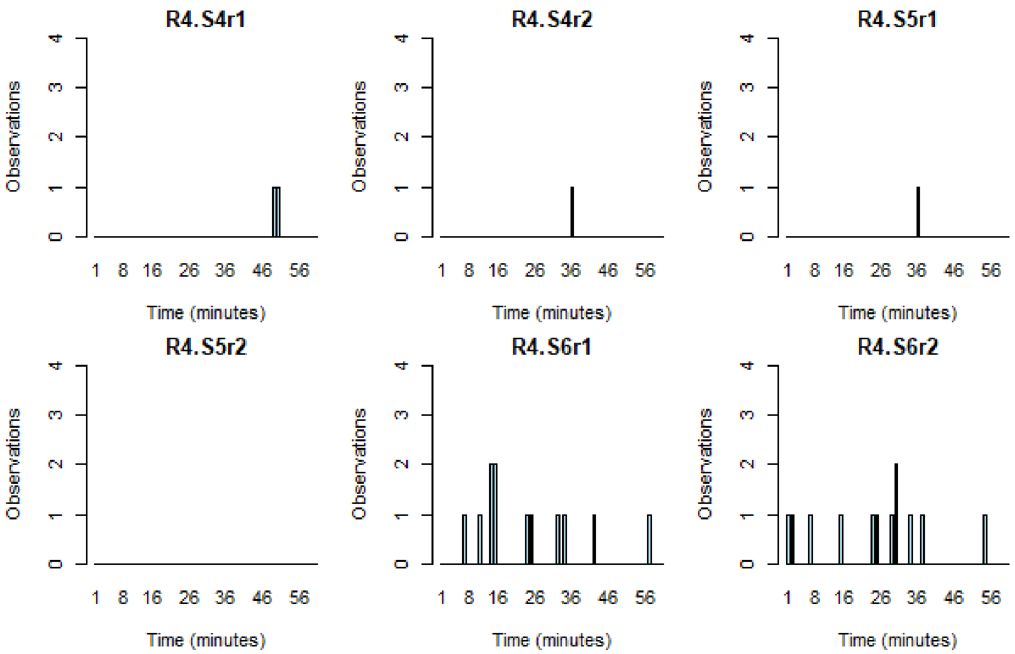


**Supplementary figure B2.** Observed items per minute for location R4, segments 4-6 (S) and replicates for that section (r).

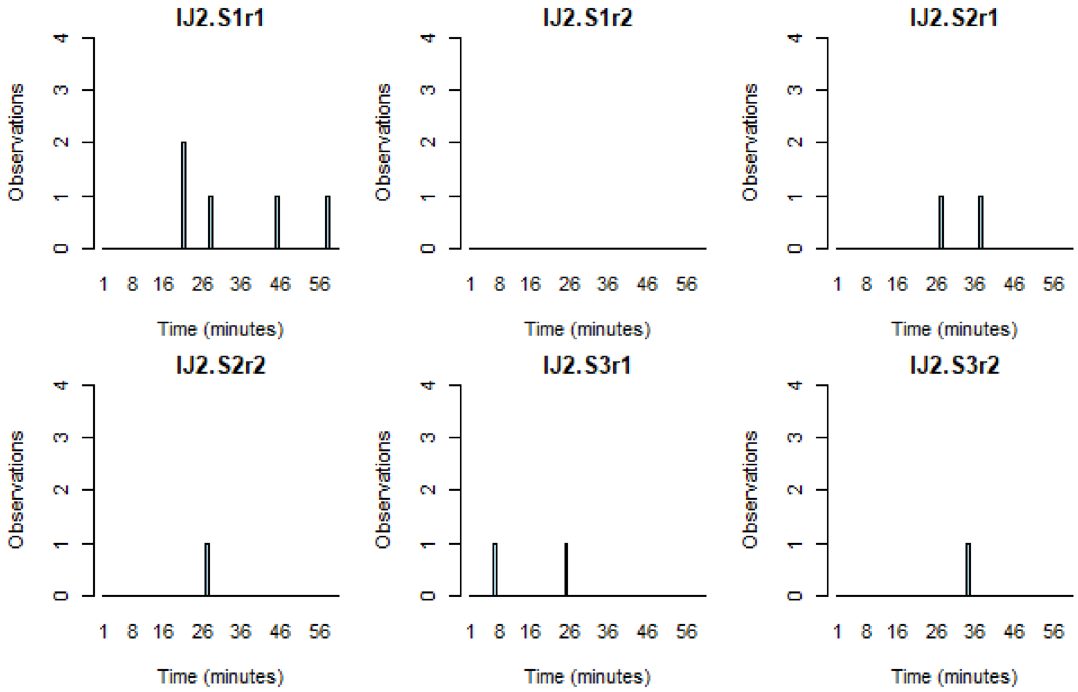


Supplementary figure B3. **Observed items per minute for location IJ2, segments 1-3 (S) and replicates for that section (r).**

**Supplementary Information C:** Note on temporal extrapolation methods

To assess the potential correlation between discharge and plastic flux, monitoring data on plastic flux was combined with discharge data obtained from Rijkswaterstaat Waterinfo[1]. For this analysis, gauging stations closest to the observation locations were selected: Sint Pieter Noord for location M1, Megen for location M2, and Lobith for location R3. Daily average discharge values were extracted for each observed day and subsequently paired with the corresponding plastic flux data. A linear regression analysis was performed to evaluate the relationship between discharge and plastic flux at each location, aiming to determine the strength and significance of the correlation.

The linear regression presented the following results for each location:

1) M1: $r^2=0.055$, RSE=239.8, F=1.105 with 1 and 19 degrees of freedom, p=0.306
2) M2: $r^2=0.009$, RSE=29.6, F=0.181 with 1 and 20 degrees of freedom, p=0.675
3) R3: $r^2=0.023$, RSE=46.38, F=0.546 with 1 and 23 degrees of freedom, p=0.468

Based on these results the relationship between discharge and microplastic flux was found to be non-significant.

[1] https://waterinfo.rws.nl/

**Supplementary Information D: Recovery rates per size + color and size + height**

**Supplementary Table D1.** Found recoveries in percentage per size class and color, including standard error and sample size. The letters in the mean column represent statistical significance results from Dunn's post-hoc test following a Kruskal-Wallis test for color and recovery (further details in supplementary table 2).

| Color | 1 $cm^2$ | 4 $cm^2$ | 9 $cm^2$ | Mean |
|---|---|---|---|---|
| Black | 19.6 ± 5.48 (13) | 62.8 ± 7.08 (14) | 61.1 ± 8.77 (14) | 48.6 ± 5.16 (41) A |
| Blue | 7.59 ± 2.32 (15) | 45.4 ± 3.50 (16) | 74.4 ± 3.50 (16) | 43.2 ± 4.39 (47) A |
| Brown | 33.7 ± 4.29 (17) | 87.1 ± 2.21 (30) | 90.4 ± 2.55 (30) | 76.6 ± 3.07 (77) B |
| Red | 15.7 ± 4.84 (15) | 61.8 ± 4.74 (16) | 85.1 ± 3.28 (16) | 55.0 ± 4.87 (47) A |
| White | 36.0 ± 5.84 (15) | 68.6 ± 4.79 (16) | 95.7 ± 3.03 (16) | 67.4 ± 4.43 (47) AB |

**Supplementary Table D2.** Found p-values in the Dunn's post-hoc test following a significant Kruskal-Wallis on the influence of particle color on recovery rate. Underlined results indicate a *P*-value below the alpha score of 0.05.

| Color | Black | Blue | Brown | Red | White |
|---|---|---|---|---|---|
| **Black** | - | 1.000 | P<0.001 | 1.000 | 0.065 |
| **Blue** | 1.000 | - | P<0.001 | 0.686 | 0.003 |
| **Brown** | P<0.001 | P<0.001 | - | 0.001 | 0.741 |
| **Red** | 1.000 | 0.686 | 0.001 | - | 0.701 |
| **White** | 0.065 | 0.003 | 0.741 | 0.701 | - |

**Supplementary Table D3.** Found recoveries in percentage per size class and height, including standard error and sample size. The letters in the mean column represent statistical significance results from Dunn's post-hoc test following a Kruskal-Wallis test for bridge height and recovery (further details in supplementary table 4).

| Height | 1 $cm^2$ | 4 $cm^2$ | 9 $cm^2$ | Mean |
|---|---|---|---|---|
| 5.4 m | 36.0 ± 4.04 (18) | 75.4 ± 3.01 (22) | 85.9 ± 3.61 (22) | 67.7 ± 3.33 (62) A |
| 14.2 m | 23.7 ± 5.34 (20) | 71.0 ± 5.54 (23) | 82.6 ± 5.49 (23) | 60.7 ± 4.38 (66) A |
| 18.6 m | 18.9 ± 4.16 (20) | 66.6 ± 4.39 (24) | 87.3 ± 2.93 (24) | 59.9 ± 4.05 (68) A |
| 20.7 m | 12.7 ± 3.97 (17) | 61.5 ± 5.34 (23) | 76.8 ± 4.88 (23) | 53.9 ± 4.32 (63) A |

**Supplementary Table D4.** Found p-values in the Dunn's post-hoc test following a significant Kruskal-Wallis on the influence of bridge height on recovery rate. Underlined results indicate a *P*-value below the alpha score of 0.05.

| Bridge height | 5.4 m | 14.2 m | 18.6 m | 20.7 m |
|---|---|---|---|---|
| **5.4 m** | - | 1.000 | 1.000 | 0.212 |
| **14.2 m** | 1.000 | - | 1.000 | 1.000 |
| **18.6 m** | 1.000 | 1.000 | - | 1.000 |
| **20.7 m** | 0.212 | 1.000 | 1.000 | - |

**Supplementary Table D5.** Results of Dunn's post-hoc test following a non-significant two-way Schreier-Ray-Hare test examining the combined effect of size+color of the particles on recovery rates. Underlined results indicate a *P*-value below the alpha score of 0.05.

| | Black.1 | Black.4 | Black.9 | Blue.1 | Blue.4 | Blue.9 | Brown.1 | Brown.4 | Brown.9 | Red.1 | Red.4 | Red.9 | White.1 | White.4 |
|---|---|---|---|---|---|---|---|---|---|---|---|---|---|---|
| **Black.4** | 0.045 | - | - | - | - | - | - | - | - | - | - | - | - | - |
| **Black.9** | 0.182 | 1.000 | - | - | - | - | - | - | - | - | - | - | - | - |
| **Blue.1** | 1.000 | 0.002 | 0.006 | - | - | - | - | - | - | - | - | - | - | - |
| **Blue.4** | 0.223 | 1.000 | 1.000 | *P*<0.001 | - | - | - | - | - | - | - | - | - | - |
| **Blue.9** | 0.001 | 1.000 | 1.000 | *P*<0.001 | 0.004 | - | - | - | - | - | - | - | - | - |
| **Brown.1** | 1.000 | 0.145 | 1.000 | 0.018 | 1.000 | *P*<0.001 | - | - | - | - | - | - | - | - |
| **Brown.4** | *P*<0.001 | 0.070 | 0.126 | *P*<0.001 | *P*<0.001 | 0.384 | *P*<0.001 | - | - | - | - | - | - | - |
| **Brown.9** | *P*<0.001 | 0.011 | 0.031 | *P*<0.001 | *P*<0.001 | 0.042 | *P*<0.001 | 1.000 | - | - | - | - | - | - |
| **Red.1** | 1.000 | 0.013 | 0.055 | 1.000 | 0.020 | 0.001 | 0.611 | *P*<0.001 | *P*<0.001 | - | - | - | - | - |
| **Red.4** | 0.011 | 1.000 | 1.000 | *P*<0.001 | 1.000 | 1.000 | 0.043 | 0.004 | 0.001 | 0.002 | - | - | - | - |
| **Red.9** | 0.001 | 1.000 | 1.000 | *P*<0.001 | *P*<0.001 | 1.000 | *P*<0.001 | 1.000 | 1.000 | *P*<0.001 | 0.109 | - | - | - |
| **White.1** | 1.000 | 0.926 | 1.000 | 0.026 | 1.000 | 0.010 | 1.000 | *P*<0.001 | *P*<0.001 | 0.782 | 0.245 | 0.001 | - | - |
| **White.4** | 0.004 | 1.000 | 1.000 | *P*<0.001 | 0.089 | 1.000 | 0.001 | 0.172 | 0.090 | 0.001 | 1.000 | 1.000 | 0.053 | - |
| **White.9** | 0.001 | 0.006 | 0.036 | *P*<0.001 | *P*<0.001 | 0.021 | *P*<0.001 | 1.000 | 1.000 | *P*<0.001 | 0.005 | 1.000 | *P*<0.001 | 0.020 |

**Supplementary Table D6.** Results of Dunn's post-hoc test following a non-significant two-way Schreier-Ray-Hare test examining the combined effect of bridge height+color of the particles on recovery rates. Underlined results indicate a P-value below the alpha score of 0.05.

| | 1-5.4 | 1-14.2 | 1-18.6 | 1-20.7 | 4-5.4 | 4-14.2 | 4-18.6 | 4-20.7 | 9-5.4 | 9-14.2 | 9-18.6 |
|---|---|---|---|---|---|---|---|---|---|---|---|
| **1-14.2** | 1 | - | - | - | - | - | - | - | - | - | - |
| **1-18.6** | 0.358 | 1.000 | - | - | - | - | - | - | - | - | - |
| **1-20.7** | 0.023 | 1.000 | 1.000 | - | - | - | - | - | - | - | - |
| **4-5.4** | *P*<0.001 | *P*<0.001 | *P*<0.001 | *P*<0.001 | - | - | - | - | - | - | - |
| **4-14.2** | 0.008 | *P*<0.001 | *P*<0.001 | *P*<0.001 | 1.000 | - | - | - | - | - | - |
| **4-18.6** | 0.006 | *P*<0.001 | *P*<0.001 | *P*<0.001 | 1.000 | 1.000 | - | - | - | - | - |
| **4-20.7** | 0.234 | 0.002 | *P*<0.001 | *P*<0.001 | 1.000 | 1.000 | 1.000 | - | - | - | - |
| **9-5.4** | *P*<0.001 | *P*<0.001 | *P*<0.001 | *P*<0.001 | 0.997 | 1.000 | 0.177 | 0.128 | - | - | - |
| **9-14.2** | 0.001 | *P*<0.001 | *P*<0.001 | *P*<0.001 | 1.000 | 1.000 | 0.339 | 0.303 | 1.000 | - | - |
| **9-18.6** | *P*<0.001 | *P*<0.001 | *P*<0.001 | *P*<0.001 | 0.408 | 1.000 | 0.066 | 0.049 | 1.000 | 1.000 | - |
| **9-20.7** | 0.001 | *P*<0.001 | *P*<0.001 | *P*<0.001 | 1.000 | 1.000 | 1.000 | 1.000 | 1.000 | 1.000 | 1.000 |
